# Integrins promote axonal regeneration after injury of the nervous system

**DOI:** 10.1111/brv.12398

**Published:** 2018-02-15

**Authors:** Bart Nieuwenhuis, Barbara Haenzi, Melissa R. Andrews, Joost Verhaagen, James W. Fawcett

**Affiliations:** ^1^ John van Geest Centre for Brain Repair, Department of Clinical Neurosciences University of Cambridge Cambridge CB2 0PY U.K.; ^2^ Laboratory for Regeneration of Sensorimotor Systems Netherlands Institute for Neuroscience, Royal Netherlands Academy of Arts and Sciences (KNAW) 1105 BA Amsterdam The Netherlands; ^3^ Biological Sciences University of Southampton Southampton SO17 1BJ U.K.; ^4^ Centre for Neurogenomics and Cognitive Research, Amsterdam Neuroscience Vrije Universiteit Amsterdam 1081 HV Amsterdam The Netherlands; ^5^ Centre of Reconstructive Neuroscience Institute of Experimental Medicine 142 20 Prague 4 Czech Republic

**Keywords:** axon regeneration, integrin, kindlin, receptor activation state, selective polarised transport, traumatic injury of the nervous system

## Abstract

Integrins are cell surface receptors that form the link between extracellular matrix molecules of the cell environment and internal cell signalling and the cytoskeleton. They are involved in several processes, e.g. adhesion and migration during development and repair. This review focuses on the role of integrins in axonal regeneration. Integrins participate in spontaneous axonal regeneration in the peripheral nervous system through binding to various ligands that either inhibit or enhance their activation and signalling. Integrin biology is more complex in the central nervous system. Integrins receptors are transported into growing axons during development, but selective polarised transport of integrins limits the regenerative response in adult neurons. Manipulation of integrins and related molecules to control their activation state and localisation within axons is a promising route towards stimulating effective regeneration in the central nervous system.

## I. INTRODUCTION

The integrin receptor family plays a role in a variety of processes including the development of various tissues (reviewed in Danen & Sonnenberg, 2003; Avraamides, Garmy‐Susini & Varner, 2008), the formation of the nervous system (reviewed in Colognato & Tzvetanova, 2011; Gardiner, 2011; Kazanis & ffrench‐Constant, 2011; Myers, Santiago Medina & Gomez, 2011), the immune response (reviewed in Means & Luster, 2010), cancer (reviewed in Guo & Giancotti, 2004; Desgrosellier & Cheresh, 2010; Schittenhelm, Tabatabai & Sipos, 2016; Paolillo, Serra & Schinelli, 2016), synaptic plasticity (reviewed in Park & Goda, 2016) and axonal regeneration in the peripheral nervous system (PNS) (reviewed in Gardiner, 2011; Eva & Fawcett, 2014). This review describes and discusses the role of integrins in axonal regeneration and their use as therapeutic targets to stimulate repair after spinal cord injury.

### (1) Structure

The structure of integrins is well characterised and has been described in many reviews (Arnaout, Goodman & Xiong, 2007; Takada, Ye & Simon, 2007; Wegener *et al*., 2007; Campbell & Humphries, 2011; Hu & Luo, 2013). Integrins are heterodimeric receptors that consist of one alpha (α) and one beta (β) subunit. In mammals, 18 α and eight β subunits have been identified giving rise to 24 unique integrin receptors (reviewed in Hynes, 2002). Integrins are type I (C‐terminus located intracellularly) glycoproteins. The ectodomain is the largest part of both the α and β subunits containing the metal‐ion and extracellular‐matrix (ECM) ligand‐binding sites. The interaction between the transmembrane domains of the subunits determines the conformation and therefore the activation state of the receptor. Inactivated integrins exist in a bent orientation as the two transmembrane domains interact closely. By contrast, activated integrins have less interaction between the transmembrane parts, resulting in a straight conformation and allowing them to bind to ligands in the ECM. The cytoplasmic tails of integrins are relatively short. They lack enzymatic activity and integrins are therefore reliant on multi‐protein complexes for signal transduction. The particularly short tail of the α subunit indicates a limited role for this subunit in intracellular processes. The cytoplasmic tail of the β subunit is also short, but contains two NPXY motifs that can interact with phosphotyrosine binding domains of intracellular proteins, such as talins (Tadokoro *et al*., 2003), kindlins (Moser *et al*., 2008; Harburger, Bouaouina & Calderwood, 2009) and various other signalling and scaffolding molecules.

### (2) Signalling

Each integrin bears a unique binding affinity for components in the heterogeneous ECM (reviewed in van der Flier & Sonnenberg, 2001; Hynes, 2002; Humphries, 2006), such as laminin, fibronectin, collagen and tenascin‐C. Importantly, integrins mediate bi‐directional signalling between the extracellular matrix and the cytoskeleton across the plasma membrane. Activated integrins bind to specific ECM ligands and induce signalling to the intracellular compartment of the cell, a process known as ‘outside‐in’ signalling. The activated integrin signalling regulates the actin cytoskeleton *via* many proteins. Firstly, talin, which interacts with the cytoplasmic tail of integrins, links them directly, or *via* vinculin, to the actin cytoskeleton. Secondly, focal adhesion kinase (FAK) is recruited to activated integrins and is a key signalling scaffold protein that activates downstream proteins such as paxillin and Src. Thirdly, integrin‐linked kinase (ILK) is another important signalling scaffold protein that phosphorylates downstream proteins. Conversely, ‘inside‐out’ signalling refers to the mechanism in which intracellular proteins bind integrins thereby inducing a conformational change that enhances the binding activity of integrins towards their ligands in the ECM, enabling intracellular signalling. Talin and kindlin, the main mediators of inside‐out signalling, are subject to various regulatory pathways that thereby affect integrin function (reviewed in Calderwood, Campbell & Critchley, 2013; Ye, Lagarrigue & Ginsberg, 2014; Rognoni, Ruppert & Fässler, 2016). Importantly, the integrin receptor family can form hundreds of protein complexes to link the ECM with the cytoskeleton. These protein complexes are also referred to as the integrin adhesome (Zaidel‐Bar *et al*., 2007; Robertson *et al*., 2015; Horton *et al*., 2015; reviewed in Winograd‐Katz *et al*., 2014; Humphries *et al*., 2015).

### (3) Integrin subunit knockouts

**Figure 1:**
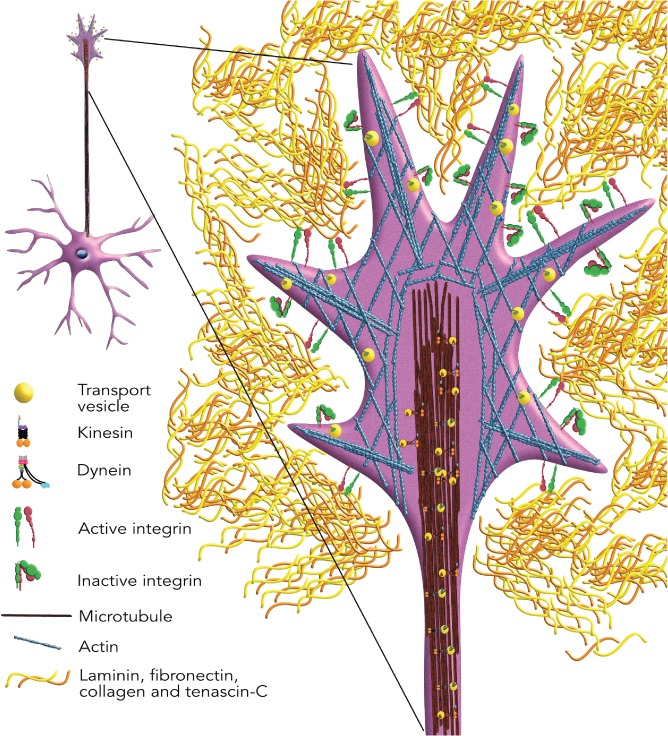
Integrins are localised to the growth cone of immature and peripheral nervous system neurons. Active and inactive integrins are present on the surface of the neuronal growth cone. However, only active integrins bind molecules of the extracellular matrix.

Whole‐system and tissue‐specific knockout studies of integrins have been fruitful in demonstrating their functional importance. It has been demonstrated in integrin‐knockout mice that integrins are important for tissue development. Depending on which integrin subunit is knocked out, the mouse phenotypes range from mild developmental defects to embryonic or perinatal lethality (reviewed in Hynes, 2002; Bouvard *et al*., 2013). The architecture and function of the nervous system is also reliant on the coordinated expression of integrin receptors and components of the ECM. Several studies examining deletion of different integrin subunits have shown varying degrees of impairment and/or changes in gross morphology, thereby confirming their fundamental role in the development and maintenance of the nervous system. For example, mutant mice carrying brain‐specific (neurons and glia) deletion of α6 integrin had abnormalities in the foliation of the cerebellum along with a reduction in process outgrowth of the Bergmann glia, yet the cerebral cortex developed normally (Marchetti *et al*., 2013). Selective deletion of αV integrin in the brain resulted in severe neurological abnormalities including seizures and ataxia as well as cerebral haemorrhage (beginning *in utero*), leading to death by 4 weeks of age in the majority of cases (McCarty *et al*., 2005). Deletion of the β1 subunit influences the majority of integrin heterodimers and not surprisingly a whole‐body knockout is embryonically lethal (Fässler & Meyer, 1995). Deletion of β1 integrins in the brain leads to death shortly after birth (Graus‐Porta *et al*., 2001) highlighting that expression of β1 integrin heterodimers in neurons and glia is essential. Several integrins have specific roles in axon regeneration that are discussed below (see Sections III and IV.1). The fact that integrins are located at the growth cone (Robles & Gomez, 2006) and respond to diverse extracellular molecular signals present in the environment of the injured PNS and central nervous system (CNS) makes them an interesting target to study axonal regeneration (Fig. 1).

## II. THE LOCALISATION OF INTEGRINS IN THE NERVOUS SYSTEM AND IMPLICATIONS FOR AXONAL REGENERATION

Integrins are expressed by every cell in the body (except red blood cells) which in the CNS includes neurons, astrocytes, microglia, oligodendrocytes, and endothelial cells (reviewed in Milner & Campbell, 2002; Schmid & Anton, 2003). Integrin function depends on the cellular localisation of the receptor. For the purposes of this review, we confine our discussion below to integrin localisation in the nervous system. Various integrins are expressed in particular sets of neurons and glia. There is also specific localisation of integrins within neurons to the somatodendritic and axonal compartments.

### (1) mRNA expression

Much of our knowledge of patterns of integrin expression comes from in situ hybridisation and reverse transcription polymerase chain reaction (RT‐PCR) studies (Table 1). In two whole‐brain expression studies, differential expression patterns of several integrin subunits were demonstrated in various brain regions. Messenger RNA (mRNA) labelling within CNS neurons varying from relatively low to significantly high levels was detected in layer V of the cortex, hippocampus (CA1, CA3 pyramidal neurons and granule neurons of the dentate gyrus), olfactory bulb, and cerebellar Purkinje neurons for α1, α2, α3, α4, α5, α6, α7, αV, β1, β3, β5, β6, and β7. Furthermore, it has been found that α8 integrin can be detected in the hippocampus and olfactory bulb (Pinkstaff et al., 1999; Chan et al., 2003). In the red nucleus, mRNA of α3, α7, αV and β1 was detected, including an up‐regulation in β1 mRNA following axotomy (Plantman et al., 2005). In addition, examination of rat dorsal root ganglia (DRGs) also revealed expression of α5, α6, α7, and β1 integrins (Wallquist et al., 2004; Gardiner et al., 2007; Gonzalez Perez et al., 2016), whereas spinal motor neurons expressed α3, α7, and β1 integrins with α6 expression appearing in these neurons after axotomy (Hammarberg et al., 2000).

**Table 1:**
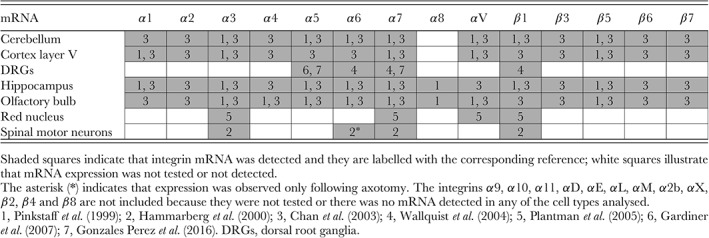
Integrin mRNA expression in the adult nervous system

### (2) Subcellular localisation

In order to assess the subcellular localisation of integrin receptors, immunohistochemical approaches or expression of labelled integrins are required. Determining whether integrins are expressed in the axonal or somatodendritic compartment is useful for understanding their potential function. In this regard, numerous studies have examined integrin expression in cultured cells, with fewer studies documenting expression in tissue sections. There are many studies demonstrating integrins in axons during embryonic development, using both immunohistochemistry and staining of cultured embryonic neurons. This is not surprising; integrins are necessary for axon growth during development (reviewed in Gardiner, 2011; Myers et al., 2011). However, in the mature CNS the picture is very different as discussed below.

Integrins have been localised within the somatodendritic compartment of adult layer V pyramidal neurons, CA1 and CA3 hippocampal neurons, granule neurons of the dentate gyrus, and Purkinje cells (Grooms, Terracio & Jones, 1993; Murase & Hayashi, 1996; Rodriguez et al., 2000; Bi et al., 2001; Schuster et al., 2001; Chan et al., 2003; Kawaguchi & Hirano, 2006; Mortillo et al., 2012) (see Table 2). Interestingly, certain integrin subunits including α3, α5 and β1 are found in the somatodendritic compartment of diverse neuronal types, which may indicate an important role in dendritic function. Other somatodendritic integrins displayed a more restricted neuron sub‐type distribution. For instance, α8 is expressed in layer V pyramidal neurons, olfactory bulb, and hippocampal neurons (Einheber et al., 1996), αV and β8 in cerebellar and hippocampal neurons (Nishimura et al., 1998; Kang et al., 2008) whereas β3 was detected in hippocampal neurons and the inner plexiform layer of the retina in addition to α5 (Kang et al., 2008; Vecino et al., 2015). Additionally, following injury, α7 and β1 subunits were found to be expressed in facial motor neurons (Kloss et al., 1999; Werner et al., 2000). The localisation of integrins in the somatodendritic compartment of adult neurons is summarised in Table 2.

**Table 2:**
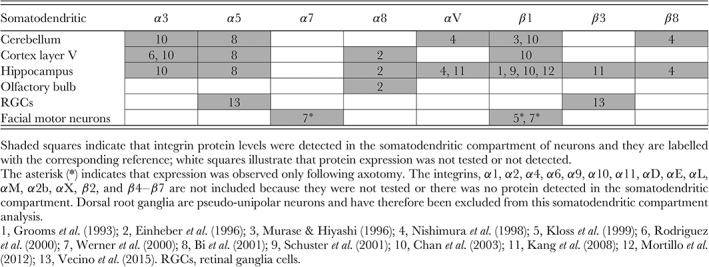
Integrins localised in the somatodendritic compartment of adult neurons

The question of whether integrins are found in axons during development and in adulthood is important to understand their function in regeneration. Very few studies however have demonstrated the presence of integrin receptors in the axonal compartment in tissue sections from the mature CNS. This is partly due to the lack of suitable antibodies, but mainly due to the down‐regulation of expression of many integrins in the adult CNS in addition to the active exclusion of integrins from most mature CNS axons as discussed below (Section VII). Some studies however have succeeded in localising endogenous integrins specifically (see Table 3); for instance α5 integrin has been found within rodent axons of layer V pyramidal neurons and reticular formation (King, McBride & Priestley, 2001; Bi et al., 2001). Interestingly, the majority of studies demonstrating axonal localisation of integrins have been in retinal ganglia cells (RGCs) and DRGs, two neuronal subtypes that have been shown experimentally to have increased regenerative capacity relative to many other CNS neuronal subtypes (Richardson & Issa, 1984; Neumann & Woolf, 1999; Leon et al., 2000; Qiu et al., 2002; Monsul et al., 2004). Within adult RGCs, α1, α3, α5, αV, and β1 subunits have been detected in axons (Hernandez, 2000; Vecino et al., 2015). α4, α5, α6, α7 and β1 subunits have been found in both processes of DRGs (Bossy, Bossywetzel & Reichardt, 1991; Yanagida, Tanaka & Maruo, 1999; Vogelezang et al., 2001; Schuster et al., 2001; Ekström et al., 2003; Wallquist et al., 2004). Overall, it appears that integrins are present in most axons during embryonic development, but in adulthood they are excluded from many CNS axons but present in retinal and sensory axons. The localisation of integrins in the axonal compartment of adult neurons is summarised in Table 3.

**Table 3:**
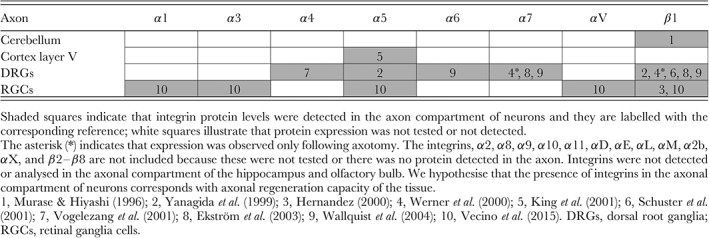
Integrins localised in the axonal compartment of adult neurons

### (3) Correlation between integrin localisation and regeneration

The neurons that have been shown to regenerate most readily are also those in which integrins are localised within axons. It is therefore interesting to link the subcellular localisation of integrins to the regenerative ability of the nervous system. As discussed above, DRGs express high levels of integrins in their axons and at least some RGC axons contain integrins (see Table 3). Furthermore, it is also known that these neurons have the capacity to regenerate successfully under certain conditions. Mature RGCs project axons through the optic nerve. These cells do not regenerate readily without intervention. However, many groups have demonstrated robust levels of axonal regeneration of RGCs following implantation of a peripheral nerve graft, generation of a lens injury, injection of zymosan (a pro‐inflammatory compound), genetic ablation of phosphatase and tensin homolog (PTEN) or suppressor of cytokine signalling 3 (SOCS3), and other interventions (So & Aguayo, 1985; Leon et al., 2000; Yin et al., 2003; Monsul et al., 2004; Park et al., 2008; P.D. Smith et al., 2009). Likewise, central projections of DRGs readily grow through crushed dorsal roots (Baer, Dawson & Marshall, 1899), but are prohibited from growing into the spinal cord through the dorsal root entry zone without interventions including implantation of a peripheral nerve graft, a (pre‐)conditioning lesion of the sciatic nerve, injection of dibutyryl cyclic AMP, or forced expression of α9 integrin among many others (David & Aguayo, 1981; Richardson & Issa, 1984; Neumann & Woolf, 1999; Qiu et al., 2002; Andrews et al., 2009). We have mentioned above that integrins are localised within the somatodendritic compartments of many cells in the brain (see Table 2), but are barely detected in the axons of Purkinje cells or within the corticospinal tract that originates from layer V cortical neurons (see Table 3). At the same time adult motor tracts are largely resistant to long‐distance regeneration in the mature CNS, presenting a major problem in promoting repair after spinal cord injury (reviewed in Case & Tessier‐Lavigne, 2005). Taken together, these data suggest that there is a strong correlation between pathways that have or retain axonal localisation of integrins and those that have the ability (albeit with growth‐promoting enhancement) to regenerate over long distances.

### (4) Integrins in the somatodendritic compartment

Discussion on the diverse function of integrins in the somatodendritic compartment is beyond the scope of this review but a recent review can be found in Park & Goda (2016). Furthermore, there is an extensive literature on the role of integrins in dendrites, spines and synapses, including participation in spine dynamics and plasticity (Rohrbough et al., 2000; Shi & Ethell, 2006; McGeachie, Cingolani & Goda, 2011; Babayan et al., 2012; Levy, Omar & Koleske, 2014; Heintz, Eva & Fawcett, 2016).

## III. INTEGRINS AND AXONAL REGENERATION IN THE PERIPHERAL NERVOUS SYSTEM

Certain integrins are up‐regulated after peripheral nerve injury (Kloss et al., 1999; Werner et al., 2000; Hammarberg et al., 2000; Wallquist et al., 2004; Gardiner et al., 2005; Gonzalez Perez et al., 2016) and can therefore be regarded as regeneration‐associated genes (reviewed in Fagoe, van Heest & Verhaagen, 2014). After injury of the peripheral nerve, the composition of the ECM changes and collagen, fibronectin and laminin become major components of the basal lamina and the endoneurium of the peripheral nerve stump distal to the lesion (reviewed in Gonzalez Perez, Udina & Navarro, 2013). Together, this creates an environment that stimulates cell adhesion and axonal regeneration (reviewed in Gardiner, 2011; Jessen, Mirsky & Arthur‐Farraj, 2015). Here, we outline the important role of integrins in promoting axonal regeneration in the injured PNS. Knockout of several integrin subunits have inhibitory effects on peripheral nerve regeneration. It is unclear whether a single knockout will prevent regeneration in the PNS, due to the presence of many integrins in the axons recognising several ligands.

### (1) Laminin‐associated integrins

Laminins are secreted by Schwann cells and are a major component of the basal lamina (Wallquist et al., 2002). They consist of α, β and γ chains that form 18 different isoforms (reviewed in Timpl & Brown, 1994; Aumailley et al., 2005; Durbeej, 2010). Many in vitro studies have shown that laminin promotes adhesion, migration and regeneration of sensory axons and Schwann cells. The laminin‐interacting integrins are α1β1, α2β1, α3β1, α6β1 and α7β1 with each bearing different affinities for the different isoforms of laminin (Table 4). The interaction of integrins and laminins was discovered in vitro by using function‐blocking antibodies as well primary cultures generated from wild type or integrin‐knockout mice that were grown on various laminin isoforms.

**Table 4:** Laminin‐associated integrins with their laminin ligands

Integrin receptor	Laminin isoform	References
α1β1	LN‐111	Condic (2001) and Desban et al. (2006)
	LN‐211/221	Colognato et al. (1997)
	LN‐511	Desban et al. (2006)
	LN‐521	Desban et al. (2006)
α2β1	LN‐111	Colognato et al. (1997)
	LN‐211/221	Colognato et al. (1997)
α3β1	LN‐111	Ivins et al. (1998) and Plantman et al. (2008)
	LN‐211/221	Tomaselli et al. (1993) and Plantman et al. (2008)
	LN‐332	Gout et al. (2001), Mechai et al. (2005) and B.E. Smith et al. (2009)
	LN‐511	Kikkawa et al. (1998) and Eble et al. (1998)
	LN‐521	Kikkawa et al. (1998)
α6β1	LN‐111	Condic & Letourneau (1997), Ivins et al. (1998); and Schöber et al. (2000)
	LN‐211/221	Delwel et al. (1994)
	LN‐332	Gout et al. (2001)
	LN‐411	Geberhiwot et al. (1999) and Plantman et al. (2008)
	LN‐511	Plantman et al. (2008)
α7β1	LN‐111	Schöber et al. (2000), Gardiner et al. (2005) and Plantman et al. (2008)
	LN‐211/221	Schöber et al. (2000) and Plantman et al. (2008)

The laminin (LN) isoforms are shown according to current laminin nomenclature (Aumailley et al., 2005). The isoforms LN‐211 and LN‐221 were assumed to be identical in the above studies and are therefore labelled LN‐211/221.

The high diversity of laminin‐associated integrins contributes to the ability of peripheral neurons to grow and regenerate on laminin‐rich areas in vivo. The laminin‐associated integrins α6β1 and α7β1 are up‐regulated in various peripheral nerve‐injury models (Table 5). A causal relationship of laminin‐associated integrins promoting regeneration was shown in mice that are deficient in α7, which exhibited reduced facial‐ (Werner et al., 2000) and sciatic nerve‐ (Gardiner et al., 2005) regeneration after axotomy. More specifically, depletion of α7 reduced axonal regeneration by 2 mm (35%) at 4 days after facial nerve crush and delayed the re‐connection of the nerve with the whisker pad compared to wild‐type mice (Werner et al., 2000). Gardiner et al. (2005) found that fewer axons in α7‐depleted mice regenerated beyond the injury site compared to controls 2 days post‐sciatic nerve crush. Another study found that inhibiting α7 and β1 function (using function‐blocking antibodies) impaired neurite outgrowth of cultured DRGs following a conditioning lesion in vivo (Ekström et al., 2003; Gardiner et al., 2005). Thus, loss of expression or function of laminin‐associated integrins results in less‐efficient regeneration of peripheral neurons. In addition, the expression of laminin‐associated integrins seems to correlate with the regenerative state of neurons. For example, neurons with a poor regenerative capacity including DRGs after a dorsal root injury (Wallquist et al., 2004), red nucleus neurons (Plantman et al., 2005), pyramidal cells and septal neurons (Werner et al., 2000) have unaltered integrin expression after axotomy.

**Table 5:** Summary of studies that assessed the expression of laminin‐associated integrins after peripheral nerve injury

Integrin receptor	Injury model	Main finding regarding integrin expression	References
α6β1	Ventral root avulsion	Up‐regulation of mRNA until 42 days after injury (2.5‐fold increase at 7 days post‐injury)	Hammarberg et al. (2000)
	Sciatic nerve transection	Up‐regulation of mRNA until 42 days after injury (2.5‐fold increase at 7 days post‐injury)	Hammarberg et al. (2000)
	Sciatic nerve transection	Up‐regulation of mRNA until 14 days after injury (3.0‐fold increase at 3 days post‐injury)	Wallquist et al. (2004)
	Sciatic nerve crush	Protein present in regenerating axons at 3 days after injury	Wallquist et al. (2004)
α7β1	Ventral root avulsion	Up‐regulation of mRNA until 42 days after injury (6.0‐fold increase at 3 days post‐injury)	Hammarberg et al. (2000)
	Facial nerve transection	Up‐regulation of protein until 42 days after injury (6.0‐fold increase at 7 days‐post injury)	Werner et al. (2000)
	Sciatic nerve transection	Up‐regulation of protein at 4 days after injury (quantification was not performed)	Werner et al. (2000)
	Sciatic nerve transection	Up‐regulation of mRNA at least 42 days after injury (ninefold increase at 14 and 21 days post‐injury)	Hammarberg et al. (2000)
	Sciatic nerve transection	Up‐regulation of mRNA until 14 days after injury (3.0‐fold increase at 3 days post‐injury)	Wallquist et al. (2004)
	Sciatic nerve transection	Up‐regulation of mRNA at 2 days after injury (2.5‐fold increase)	Gonzalez Perez et al. (2016)
	Sciatic nerve crush	Protein present in regenerating axons at 3 days after injury	Wallquist et al. (2004)
	Sciatic nerve crush	Up‐regulation of protein for at least 14 days in medium‐ to large‐diameter (NF200 positive) dorsal root ganglion neurons and to a lesser extent in smaller peptidergic neurons. No expression in smaller non‐peptidergic neurons	Gardiner et al. (2005)

### (2) Fibronectin‐associated integrins

Fibronectin is another important component of the ECM that stimulates the pro‐regenerative state of PNS neurons. Fibronectin is a large glycoprotein that consists of two subunits which form a dimer (reviewed in Singh, Carraher & Schwarzbauer, 2010; Schwarzbauer & DeSimone, 2011). Fibronectin is secreted mainly by fibroblasts (Zhu et al., 2015) but also by astrocytes and Schwann cells (Baron‐Van Evercooren et al., 1986; Egan & Vijayan, 1991; Tom et al., 2004). Fibronectin is enriched in the injured PNS and contributes to an environment that is permissive for integrin‐mediated adhesion and regeneration. Integrins bind to fibronectin via an Arg‐Gly‐Asp (RGD) domain, which is also found on other matrix molecules such as tenascin‐C and some laminins.

Fibronectin‐associated integrins in adult neurons include α4β1, α5β1, α8β1 and αV integrins. α4β1 binds to fibronectin, however its main role is as a thrombospondin and osteopontin receptor and as a vascular cell adhesion molecule (VCAM) receptor in inflammatory cells. α4β1 and α5β1 integrins are expressed at high levels in native DRG neurons and growth cones of regenerating neurons (Lefcort et al., 1992; Mathews & ffrench‐Constant, 1995; Yanagida et al., 1999; Vogelezang et al., 2001; Hu & Strittmatter, 2008; Saunders et al., 2014). Several studies have shown that the expression of fibronectin‐associated integrins is enhanced acutely after injury. α5β1 mRNA expression levels were shown to double in DRGs and spinal cord at 2 days post‐sciatic nerve transection (Gonzalez Perez et al., 2016), but were found to remain unaltered 7 days post‐sciatic nerve crush (Gardiner et al., 2007). At longer time points after injury, a few studies suggest that there are changes in the localisation of integrins. For instance, the localisation of α5β1 was targeted towards the growth cones favouring neurite elongation of cultured preconditioned DRG neurons (Gardiner et al., 2007). Consistently, α4β1 has been detected at the growth cones in vivo while expression levels were unaltered at 4 days after a sciatic nerve injury (Vogelezang et al., 2001).

The pro‐regenerative phenotype of fibronectin‐associated integrins has been investigated in vitro. PC12 cells, that grow poorly on fibronectin, were shown to express α5β1 at low levels and α4β1 not at all (Tomaselli, Damsky & Reichardt, 1987; Vogelezang et al., 2001). However, cells engineered to express α4β1 showed a 2.5‐fold increase in outgrowth on fibronectin compared to controls, indicating that α4β1 expression promotes neurite growth on fibronectin (Vogelezang et al., 2001, 2007). The regenerating effects of α5β1 on a fibronectin substrate was first shown when it was overexpressed in vitro in adult DRGs that had a roughly threefold increase in neurite count and length on fibronectin compared to controls (Condic, 2001). Taken together, both α4β1 and α5β1 enhance neurite outgrowth on fibronectin in vitro. There are no reports on axonal regeneration experiments in transgenic mice that lack α4 or α5 because these animals are not viable (Yang, Rayburn & Hynes, 1993, 1995).

### (3) Collagen‐associated integrins

Collagen is another ECM molecule that is highly up‐regulated after peripheral nerve injury and is synthesised by Schwann cells and fibroblasts (reviewed in Koopmans, Hasse & Sinis, 2009). The high amount of collagen at the injury site could indicate an important role for axonal integrins that interact with collagen. The collagen‐associated integrins expressed by neurons are α1β1 (Ivins, Yurchenco & Lander, 2000; Vecino et al., 2015), α2β1 (Bradshaw et al., 1995; Emsley et al., 2000; Khalsa et al., 2000), and αVβ8 (Venstrom & Reichardt, 1995; Nishimura et al., 1998). α10β1 and α11β1, two other collagen‐associated integrins, are not expressed in the nervous system. The neuronal collagen‐associated integrins have been shown to contribute to neurite outgrowth on collagen in cell cultures (Bradshaw et al., 1995; Venstrom & Reichardt, 1995; Ivins et al., 2000; Vecino et al., 2015). However, to our knowledge, there are no reports on manipulation of collagen‐associated integrins after injury in vivo. It would therefore be interesting to explore whether activation or overexpression of the collagen‐associated integrins is beneficial for regeneration in the PNS.

In summary, peripheral nerve injury leads to an up‐regulation of many ECM molecules including laminin, fibronectin and collagen. Neurons in the PNS express many of the integrins that respond to this post‐injury ECM environment, which contributes to the spontaneous regeneration observed after peripheral nerve injury. Thus, studies in the PNS have shown that matching the ECM environment with the appropriate integrin expression pattern promotes axonal regeneration of mature neurons. It is therefore reasonable to try the same approach in the CNS and promote regeneration via integrin overexpression.

## IV. INTEGRINS THAT BIND TO TENASCIN‐C PROMOTE AXONAL REGENERATION IN THE CENTRAL NERVOUS SYSTEM

### (1) Tenascin‐C‐associated integrins

Tenascin‐C is a ligand for integrins (reviewed in Tucker & Chiquet‐Ehrismann, 2015) and is predominantly expressed in the CNS during development. However, injury results in a steep up‐regulation of this ECM glycoprotein by reactive astrocytes (reviewed in Silver & Miller, 2004; Gervasi, Kwok & Fawcett, 2008; Wiese, Karus & Faissner, 2012). Tenascin‐C is enriched within and surrounding the glial scar after spinal cord injury (Zhang et al., 1997; Tang, Davies & Davies, 2003; Andrews et al., 2009), and it is expressed at the dorsal root entry zone after a dorsal root injury (Andrews et al., 2009; Cheah et al., 2016). Tenascin‐C is expressed not only by astrocytes but also by fibroblasts and spinal neurons among others (Zhang et al., 1995, 1997; Tang et al., 2003; Zhang et al., 2015). Thus, tenascin‐C is enriched at the site of injury which regenerating axons have to penetrate in order to reconnect to their target tissue. Therefore, tenascin‐C is a promising target to promote axonal regeneration after CNS trauma.

The tenascin‐C‐associated integrins include α2β1 (Sriramarao, Mendler & Bourdon, 1993; Schaff et al., 2011), α7β1 (Mercado et al., 2004), α8β1 (Schnapp et al., 1995; Varnum‐Finney et al., 1995; Denda, Reichardt & Müller, 1998) and α9β1 (Yokosaki et al., 1994, 1998). They are expressed in developing neurons and most of them recognise the fibronectin type 3 repeat domain of tenascin‐C through its RGD attachment site. α9β1 is an exception as it recognises a different sequence in this domain, AEIDGIEL (Yokosaki et al., 1998). Tenascin‐C‐associated integrins have been shown to be required for neurite outgrowth, as assessed in experiments with function‐blocking antibodies in vitro (Varnum‐Finney et al., 1995; Mercado et al., 2004; Andrews et al., 2009). Providing that neurons express an appropriate integrin, tenascin‐C is a substrate that favours neurite outgrowth and axonal regeneration (Götz et al., 1996; Rigato et al., 2002; Chen et al., 2009; Liu et al., 2010; Yu et al., 2011), but for neurons lacking the appropriate receptors tenascin‐C is inhibitory (reviewed in Faissner, 1997). Adult CNS neurons do not express tenascin‐C‐binding integrins within their axons, even after injury (Pinkstaff et al., 1999; Andrews et al., 2009). Although glial cell types retain the ability to interact with tenascin‐C, it is anti‐adhesive to most adult neurons due to their lack of expression of tenascin‐C‐binding integrins (Zhang et al., 1995; Golding et al., 1999). Thus, up‐regulation of tenascin‐C results in an anti‐adhesive and growth‐inhibiting environment for neurons in the CNS. In the next section, we will discuss experiments that show that tenascin‐C is only an axon‐regeneration ligand in the injured adult CNS when neurons are engineered to express an appropriate integrin, such as α9β1.

### (2) Viral vector‐mediated delivery of α9 integrin in dorsal root ganglia promotes sensory axon regeneration in the central nervous system

We hypothesised that low or absent integrin expression in CNS axons (see Table 3) contributes to the poor regenerative capacity of most CNS neurons. To achieve regeneration in the CNS, expression of tenascin‐C‐binding integrins in neurons might provide a promising tool to overcome the tenascin‐C‐rich injury site. Viral vector‐mediated delivery of α9 into DRGs results in integrin localisation in the axon and could therefore induce integrin‐mediated axonal regeneration (Andrews et al., 2009, 2016; Cheah et al., 2016). Indeed, exogenous expression of α9 allowed cultured adult DRGs to extend neurites on tenascin‐C substrates in vitro, while neurite outgrowth was largely absent in controls (Andrews et al., 2009). Furthermore, in vivo reintroduction of α9 in DRGs improved sensory axonal regeneration into tenascin‐C‐rich regions after a dorsal root injury or dorsal column crush lesion (Andrews et al., 2009). However, regeneration was limited to the lesion site; there was no axonal growth extending beyond the lesion. Nevertheless, this was enough to result in limited sensory recovery (Andrews et al., 2009). These results demonstrate that tenascin‐C‐associated integrins such as α9β1 are a viable target to promote axonal regeneration in the CNS. However, this approach should be combined with additional factors, such as integrin activators, to promote long‐distance regeneration as well as functional recovery in vivo. Section V will demonstrate that integrins become inactivated by stimuli of the extracellular environment, and thus methods that target the activation of the receptor (discussed in Section VI) could enhance axonal regeneration (discussed in Section VI.3c).

## V. INTEGRINS BECOME INACTIVATED AT THE LESION SITE AFTER CENTRAL NERVOUS SYSTEM INJURY

**Figure 2:**
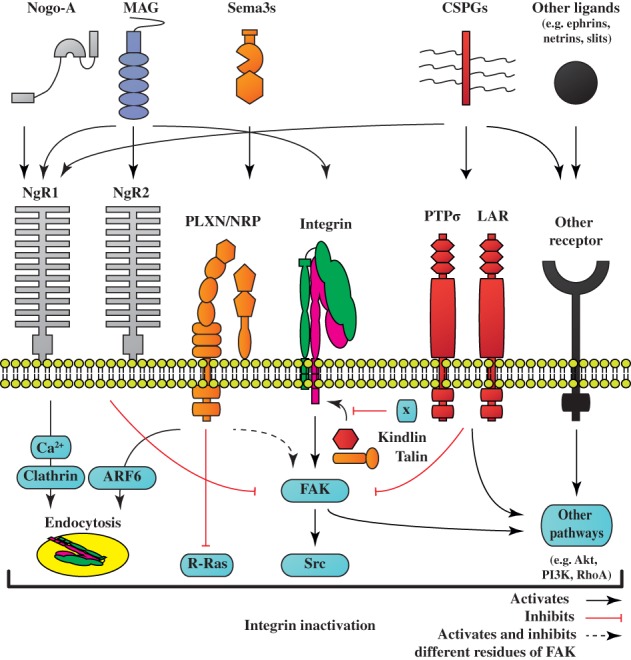
Molecular mechanisms of integrin inactivation after trauma in the nervous system. Integrins at the growth cones of regenerating axons are exposed to the extracellular environment of the lesion site. Integrins recruit focal adhesion kinases (FAKs) among others, which in turn, activate downstream signalling molecules such as protein kinase B (Akt3), phosphoinositide 3‐kinase (PI3K), Ras homolog gene family member A (RhoA), and Src kinase. However, most integrins exist in a bent, inactive state at the cell surface. The lesion site is rich in axon‐repulsive molecules, including Nogo‐A, myelin‐associated glycoprotein (MAG), class III semaphorins (Sema3s), and chondroitin sulphate proteoglycans (CSPGs). These molecules bind to several receptors, such as leukocyte common antigen‐related phosphatase (LAR), Nogo receptors (NgR1, NgR2), the plexin/neuropilin (PLXN/NRP) complex and protein tyrosine phosphatase σ (PTPσ), to suppress integrin signalling and axon regeneration. Nogo‐A binds to NgR1 and inhibits the phosphorylation of FAK. MAG is a direct ligand for integrins and stimulates integrin signalling. However, MAG also has an opposing effect by NgRs signalling that indirectly elevates intracellular calcium levels and stimulates clathrin‐mediated endocytosis of integrins. Most Sema3s mediate signalling via the PLXN/NRP receptor complex that results in inactivation of R‐Ras, which in turn interferes with integrin signalling, and activates ADP‐ribosylation factor 6 (ARF6) to remove integrins from the cell surface. Sema3A signalling results in the phosphorylation (Tyr397, Tyr576, Tyr577, Tyr925) and de‐phosphorylation (Tyr407, Tyr861) of different residues of FAK for Sema3A‐mediated axonal remodelling. CSPGs interact with many receptors, including LAR, NgR1 and PTPσ. The CSPG aggrecan has been shown to reduce FAK signalling, but the exact mechanisms remain to be identified. Other ligands such as ephrins, netrins and slits are also known to interfere with integrin signalling. In addition, there is evidence that integrin activation by kindlins and talins is inhibited by various regulatory mechanisms (illustrated as x).

Axon‐repulsive molecules at the injury site, such as chondroitin sulphate proteoglycans (CSPGs) (reviewed in Kwok et al., 2011), myelin‐derived molecules (reviewed in Alizadeh, Dyck & Karimi‐Abdolrezaee, 2015; Boghdadi, Teo & Bourne, 2017) and classical repulsive axon‐guidance molecules (reviewed in de Wit & Verhaagen, 2003; Giger, Hollis & Tuszynski, 2010; Hollis, 2015) have a broad range of functions. Here we highlight that most axon‐repulsive molecules initiate inactivation of integrins (see Fig. 2).

### (1) Nogo‐A

Nogo‐A is a myelin‐derived axon repulsive molecule that restricts axonal regeneration after CNS injury (Schnell & Schwab, 1990; Bregman *et al*., 1995; Brösamle *et al*., 2000; Kim *et al*., 2003; Simonen *et al*., 2003; Zheng *et al*., 2003; Sicotte *et al*., 2003; Dimou *et al*., 2006; Cafferty & Strittmatter, 2006; Lee *et al*., 2010; Wang *et al*., 2015). Nogo receptor 1 (NgR1) is a glycosylphosphatidylinositol (GPI)‐linked molecule, and was the first receptor identified for Nogo proteins (Fournier, GrandPre & Strittmatter, 2001). NgR1 has been shown to transduce Nogo signalling across the plasma membrane by interacting with several other receptors such as leucine‐rich repeat and immunoglobulin‐like domain‐containing Nogo receptor‐interacting protein (Lingo‐1), p75, and Troy (Wang *et al*., 2002; Mi *et al*., 2004; Park *et al*., 2005; Shao *et al*., 2005). Interestingly, Nogo‐A has been shown to suppress integrin signalling through integrin inactivation *in vitro* (Hu & Strittmatter, 2008; Tan *et al*., 2011) and *in vivo* (Huo *et al*., 2015). Specifically, it has been shown in cell lines that Nogo‐A interferes with the function of fibronectin‐associated integrins α4β1, α5β1 and αVβ3, but not laminin‐associated integrin α6β1 (Hu & Strittmatter, 2008). Nogo‐A's attenuation of DRGs neurite outgrowth *in vitro* has been consistently greater on fibronectin than on laminin (Hu & Strittmatter, 2008). Further, it has been shown *in vivo* after an optic nerve crush that Nogo‐A down‐regulates the expression of αV integrins and thereby reduces integrin signalling, in this case the phosphorylation of FAK (Huo *et al*., 2015). The same study showed that the expression of another fibronectin‐associated integrin, α5, was unaltered by Nogo‐A in the injured optic nerve suggesting that Nogo‐A has varied effects on different fibronectin‐associated integrins, perhaps dependent on the function of the integrin. Taken together, both studies suggest that Nogo‐A inhibits specific integrin signalling by inactivation and internalisation (Hu & Strittmatter, 2008; Huo *et al*., 2015). However, the mechanisms that dictate the interaction between Nogo proteins and integrins require further investigation.

### (2) Myelin‐associated‐glycoprotein

Myelin‐associated‐glycoprotein (MAG) is another myelin‐derived axon‐repulsive molecule (Mukhopadhyay *et al*., 1994; McKerracher *et al*., 1994; Schäfer *et al*., 1996). MAG binds to NgR1 (Domeniconi *et al*., 2002; Wang *et al*., 2002; Liu *et al*., 2002; Laurén *et al*., 2007) and NgR2 (Venkatesh *et al*., 2005) and many other neuronal receptors (Wong *et al*., 2002; Atwal *et al*., 2008; Stiles *et al*., 2013). It has been known for more than two decades that MAG antagonises integrin signalling and function (Bachmann *et al*., 1995). More recently, the underlying mechanism became clearer when it was shown that MAG is axon repulsive in cultured postnatal hippocampal neurons and cerebellar granule cells by modulating integrin‐signalling independently of NgRs (Goh *et al*., 2008). This study found that β1 integrin is a direct receptor of MAG and led to increased phosphorylation of FAK. This result is unexpected since FAK signalling is associated with axonal growth. It may therefore be that the signalling is only locally affected and shifts to sites of axon attraction at the growth cone, where new integrin adhesion complexes form to initiate axon guidance. This asymmetrical signalling hypothesis is supported by a study that showed that a local MAG gradient removed integrins at the site of the MAG source only, while untreated neurons had a symmetric distribution of integrins at the growth cone (Hines, Abu‐Rub & Henley, 2010). MAG signalling has also been shown to initiate changes in intracellular Ca^2+^, thereby inducing clathrin‐mediated endocytosis of integrins from the growth cones of *Xenopus laevis* spinal neurons (Hines *et al*., 2010). Taken together, MAG mediates its axon‐repulsive effects by modulating integrin signalling, partly through direct interaction and partly through another signalling complex most likely including NgRs that cause Ca^2+^‐dependent internalisation of integrins.

### (3) Aggrecan

Aggrecan is one of the CSPGs produced by neurons and astrocytes, and is present in the scar tissue that restricts axonal regeneration (Lemons *et al*., 2003; reviewed in Silver & Miller, 2004). Not surprisingly, adult DRG neurons have restricted neurite outgrowth when cultured on substrates that contain the glycan chains of CSPGs (Tom *et al*., 2004; Steinmetz *et al*., 2005). Aggrecan has been shown to cause a temporary but rapid decrease in integrin‐mediated phosphorylation of FAK, and a long‐term decrease of Src phosphorylation which is downstream of FAK, leading to inhibition of DRG neurite outgrowth (Tan *et al*., 2011). The molecular mechanism of how aggrecan inhibits integrin signalling is currently unknown. However, it is known that aggrecan does not affect the number of integrin receptors at the plasma membrane (Tan *et al*., 2011). Thus, it interferes with integrin signalling independent of receptor endocytosis. It may interfere indirectly with integrin signalling *via* activation of CSPG receptors such as protein tyrosine phosphatase σ (PTPσ) (Shen *et al*., 2009; Fry *et al*., 2010), leukocyte common antigen‐related phosphatase (LAR) (Fisher *et al*., 2011; Xu *et al*., 2015) or the Nogo receptors NgR1 and NgR3 (Dickendesher *et al*., 2012).

### (4) Class III semaphorins

Class III semaphorins (Sema3s) are classical axon‐guidance molecules that are mainly produced by migrating fibroblasts, pericytes and vascular cells in the core of the scar (Pasterkamp, Giger & Verhaagen, 1998; Pasterkamp *et al*., 1999; de Winter *et al*., 2002; Tannemaat *et al*., 2007; Mire *et al*., 2008; Minor *et al*., 2011). It has been shown that Sema3s restrict axonal regeneration after spinal cord injury (Kaneko *et al*., 2006; Mire *et al*., 2008; Lee *et al*., 2010; Minor *et al*., 2011; reviewed in Mecollari, Nieuwenhuis & Verhaagen, 2014). Most Sema3s interact with neuropilins (NRPs), while signal transduction is mediated *via* the plexin (PLXN) co‐receptor (reviewed in Sharma, Verhaagen & Harvey, 2012). The pleiotropic NRPs have also been shown to interact with integrins (Fukasawa, Matsushita & Korc, 2007; Valdembri *et al*., 2009) which could suggest that Sema3s affect integrin signalling *via* NRPs. Nonetheless, it has been shown that PLXN signalling leads to rapid disassembly of integrin adhesion at the cell surface and causes actin depolymerisation in various non‐neuronal cell lines (Barberis *et al*., 2004). It has been observed in cultured cortical‐ and hippocampal neurons that Sema3A‐induced collapse of growth cones requires FAK signalling downstream of integrins (Bechara *et al*., 2008; Chacón, Fernández & Rico, 2010). More specifically, Sema3A resulted in the phosphorylation (Tyr397, Tyr576, Tyr577, Tyr925) and de‐phosphorylation (Tyr407, Tyr861) of different residues of FAK, confirming the central signalling role of this kinase, and this may result in the activation and inhibition of several signalling pathways to induce growth‐cone collapse (Chacón *et al*., 2010). The strongest evidence that Sema3s regulate the activation of integrins originates from studies of angiogenesis. Sema3s, except for Sema3C, inhibit integrin signalling in blood vessels (Table 6). Sakurai *et al*. (2010) highlighted two mechanisms by which Sema3E signalling *via* PLXN reduces the function of integrins in endothelial cells: (*i*) activation of endosomes that contain ADP‐ribosylation factor 6 (ARF6) removes integrins from the cell surface; and (*ii*) inactivation of R‐Ras GTPases, which normally activate integrins (Zhang *et al*., 1996; Keely *et al*., 1999; Wang *et al*., 2000; Ivins *et al*., 2000; Self *et al*., 2001) *via* the phosphoinositide 3‐kinase (PI3K) signalling pathway (Berrier *et al*., 2000; Oinuma *et al*., 2010). Sema3s could exert the same and other integrin‐mediated mechanisms in neurons resulting in axon repulsion after CNS injuries.

**Table 6:** Summary of studies in the field of angiogenesis that show that class III semaphorins (Sema3s) modulate integrins

Sema3s	Main finding regarding integrins after Sema3 overexpression	References
Sema3A	Inhibiting the signalling of αIIbβ3 *in vitro*	Kashiwagi *et al*. (2005)
	Inhibiting the activation of β1 *via* NRP1/PLXN *in vivo*	Serini *et al*. (2003)
Sema3C	Phosphorylation of β1, but not FAK, *via* NRP/PLXN *in vitro*	Banu *et al*. (2006)
Sema3E	Inhibiting the activation of integrins by inactivation of R‐Ras *in vitro*	Sakurai *et al*. (2010)
	Endocytosis of integrins by activation of ARF6‐postive vesicles *in vitro*	Sakurai *et al*. (2010)
Sema3F	Inhibiting the activation of β1 *via* NRP1/PLXN *in vivo*	Serini *et al*. (2003)

ARF6, ADP‐ribosylation factor 6; FAK, focal adhesion kinase; NRP, neuropilin; PLXN, plexin; R‐Ras, Ras‐related protein R‐Ras.

Taken together, a variety of molecules in the scar and lesion milieu have the ability to regulate integrin function (Fig. 2). These molecules affect integrin binding to their ECM ligands and thereby subsequent downstream signalling as well as integrin levels at the cell surface by endocytosis. Integrins are not the only receptors and ligands affecting growth and regeneration. There are other signalling cascades that feed positively or negatively into integrin downstream signalling. For instance, molecules such as protein kinase B (Akt), Ras homolog gene family member A (RhoA) and PI3K are regulated by many receptors. Finally, another level of control is the pathways that influence integrin activation through kindlins and talin. Studying integrin inhibition has revealed integrin‐specific and general mechanisms whereby axonal regeneration fails in adult CNS neurons. Inactivation of integrins in the injured spinal cord also explains the modest axonal regeneration that was observed after forced expression of α9 *in vivo* (Andrews *et al*., 2009). Expression of an appropriate integrin and overcoming integrin inactivation could therefore be a general approach to promote axonal regeneration in the CNS.

## VI. INTEGRIN ACTIVATORS PROMOTE SENSORY AXONAL REGENERATION IN THE SPINAL CORD

**Figure 3:**
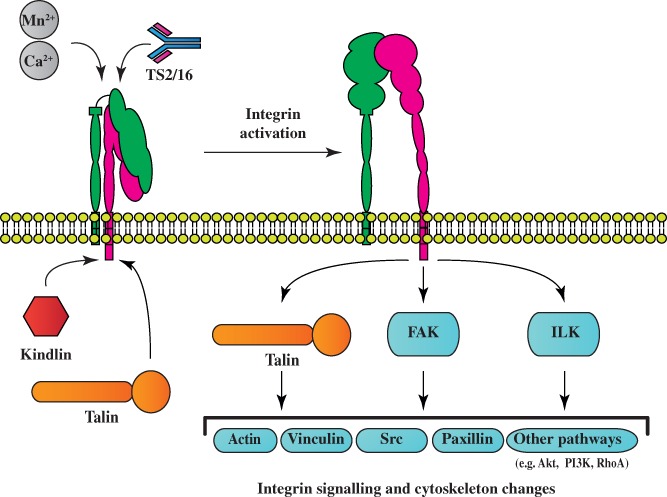
Molecular mechanisms for integrin activation. Integrins exist in two activation states on the cell surface: a bent inactive and a straight active state. There are several ways to activate integrins: (i) cations such as Ca^2+^ and Mn^2+^ interact with a metal ion‐binding site at the ectodomain of the integrin to activate the receptor; (ii) kindlins and talins are two families of intracellular proteins that bind to the cytoplasmic tail of β1 integrins to activate the heterodimeric complex; (iii) the monoclonal antibody TS2/16 binds to the ectodomain of human β1 integrins to induce a conformational change and receptor activation. Activated integrins have their ectodomain exposed and bind extracellular matrix ligands, which leads to intracellular signalling and changes of the cytoskeleton. Activation of certain integrins can result in cell adhesion and axonal regeneration. FAK, focal adhesion kinase; ILK, integrin‐linked kinase; PI3K, phosphoinositide 3‐kinase.

Integrins need to be in their active state to interact with components of the ECM and thereby induce an increase in neurite outgrowth and axonal regeneration. Once activated they stimulate FAK and other downstream signalling molecules that are essential for growth cone dynamics and axonal guidance (Robles & Gomez, 2006; reviewed in Mitra, Hanson & Schlaepfer, 2005). Here we discuss the best‐characterised integrin activators with regard to axonal regeneration (see Fig. 3).

### (1) Manganese

Manganese (Mn^2+^) is widely used in *in vitro* experiments to enhance the ligand‐binding affinity of integrins to the ECM. Divalent cations such as Ca^2+^ and Mn^2+^ interact with metal‐ion binding sites of the α integrin subunit and facilitate integrin signalling (Mould, Akiyama & Humphries, 1995; Oxvig & Springer, 1998). This ‘outside‐in’ activation of integrins by Mn^2+^ has been shown to increase neurite outgrowth in various neuronal cell culture assays (Ivins *et al*., 2000; Lein *et al*., 2000; Lemons & Condic, 2006; Tan *et al*., 2011). Importantly, activation of integrins has been shown to reverse the growth‐inhibitory effects of Nogo‐A and aggrecan in cultured DRG neurons (Tan *et al*., 2011). Recently, Mn^2+^ has also been shown to abolish ephrinA3‐mediated collapse of proximal dendritic spines in Purkinje cells *via* integrin activation *in vitro* (Heintz *et al*., 2016). Thus, it is possible to reverse integrin inactivation with Mn^2+^ treatment *in vitro*. However, Mn^2+^ is not suitable for *in vivo* studies because excess and long‐term exposure to Mn^2+^ causes neuronal toxicity (reviewed in Guilarte, 2013).

### (2) Integrin‐activating antibodies

Another classic approach to activate integrins is using antibodies that bind selectively to the ligand‐binding region of activated β1 integrin, which can be used both for detecting activated integrins and for maintaining them in the activated state (Takada & Puzon, 1993; Takagi *et al*., 1997); these antibodies are mostly effective on human integrins. The anti‐β1‐activating monoclonal antibody TS2/16 interacts with all human integrin heterodimers that contain β1 and less strongly with rodent β1, regardless of the α subunit (Tsuchida *et al*., 1997). Due to the wide spectrum of integrins that can be targeted, the antibody TS2/16 is particularly interesting and has been used in outgrowth assays. For example, TS2/16‐mediated activation of integrins has been shown to reverse the inhibitory effects of Nogo‐A on a human T‐lymphocyte cell line grown on fibronectin (Hu & Strittmatter, 2008) as well as to inhibit the effects of aggrecan on axon growth of motor neurons that were derived from human embryonic stem cells (Tan *et al*., 2011). Thus, the TS2/16 antibody reverses axon‐repulsive effects of molecules such as Nogo‐A and aggrecan. However, a limitation of applying integrin antibodies is that these need frequent or continuous delivery *in vivo*. In addition, masking of epitopes due to integrin interactions with ECM ligands can reduce the efficiency of integrin‐binding antibodies (Mould *et al*., 2016).

### (3) Intracellular proteins

The kindlins and talins are two families of intracellular proteins that bind to the cytoplasmic tail of β integrins and activate the heterodimeric receptor. Integrin activation is ubiquitous throughout the body, but the exact mechanism of the ‘inside‐out’ activation by kindlin and talin is subject to intense debate (reviewed in Meves *et al*., 2009; Moser *et al*., 2009; Shattil, Kim & Ginsberg, 2010; Campbell & Humphries, 2011; Calderwood *et al*., 2013; Eva & Fawcett, 2014). Despite the limited number of studies investigating the role of these molecules in the nervous system, they have been utilised to enhance integrin‐ligand binding and axonal outgrowth of neurons (Tan *et al*., 2012, 2015; Dingyu *et al*., 2015; Cheah *et al*., 2016) as discussed below.

#### (a) *Talins*

Talin isoforms 1 and 2 are expressed in the nervous system (Monkley, Pritchard & Critchley, 2001; Senetar, Moncman & McCann, 2007; Debrand *et al*., 2009; Tan *et al*., 2015). In nerve growth factor‐stimulated PC12 cells, overexpression of the full‐length and constitutively activated isoforms of talin has been shown to promote neurite outgrowth in the presence of the repulsive extracellular matrix protein aggrecan (Tan *et al*., 2015). Dingyu *et al*. (2015) examined the structural tensions of the cytoskeleton in this cell line by fluorescence resonance energy transfer (FRET) imaging and application of genetically encoded optical force probes. They found that CSPGs including aggrecan reduce intracellular structural forces and that overexpression of full‐length talin rescued these tensions. In addition, talin decreased the phosphorylation of Rho‐associated protein kinase 1 (ROCK1) and increased the activation of extracellular signal‐related kinase (ERK) and FAK proteins (Dingyu *et al*., 2015). Based on these results *in vitro*, full‐length talin could be a valuable activator of integrins to reverse the effects of the axon‐repulsive molecules that are present in the injured spinal cord. However, the large size of the full‐length protein presents a challenge for talin expression in neurons. In studies using primary cultures of DRG neurons, only the talin head domain has been overexpressed (Tan *et al*., 2015). The talin‐head domain is required to interact with the cytoplasmic tail of the β integrin subunit and to activate the heterodimeric receptor (García‐Alvarez *et al*., 2003; Tadokoro *et al*., 2003; Wegener *et al*., 2007). However, the talin head domain alone acted as a dominant negative for endogenous talin, and DRG neurite outgrowth on laminin and on aggrecan–laminin substrates was reduced (Tan *et al*., 2015). Based on these results, the talin head domain alone is not suitable to promote integrin signalling. The limited effect of the talin head is possibly due to the endogenous expression of full‐length talins in neurons or because the rod domain is required to link integrins directly with the cytoskeleton. Another disadvantage of talin‐targeted experiments and therapeutics is the fact that full‐length talins are so large that they are not suitable for an adeno‐associated viral vector (AAV)‐based gene‐delivery approach. The coding sequence for talin is roughly 7500 base pairs, which exceeds the AAV packaging limit of approximately 4700 base pairs. Taken together, talin overexpression would be a promising target to enhance axonal regeneration since it enables integrin signalling directly to the cytoskeleton but is not feasible with the AAV technologies currently available. Talin itself is subject to several regulatory influences, which in turn affect integrin activation and function (reviewed in Ye *et al*., 2014).

#### (b) *Kindlins*

There are three isoforms of kindlin: kindlin‐1, kindlin‐2 and kindlin‐3. Localisation of the kindlin isoforms in the nervous system is described here. Kindlin‐1 is not expressed by cells of the nervous system (Ussar *et al*., 2006; Tan *et al*., 2012), but is primarily found in epithelial cells (Lai‐Cheong *et al*., 2008; Ussar *et al*., 2008). Kindlin‐2 is ubiquitously expressed throughout the body (Ussar *et al*., 2006) and *in situ* hybridisation and RT‐PCR studies on whole‐brain lysate demonstrated that kindlin‐2 mRNA is present in the brain, while kindlin‐1 and kindlin‐3 were not detected (Ussar *et al*., 2006; Tan *et al*., 2012). Immunochemistry on cultured cells confirmed that this isoform is expressed by neurons including DRGs, RGCs, and hippocampal and Purkinje neurons (Tan *et al*., 2012). In these cultures, kindlin‐2 was also found in non‐neuronal cells like astroctyes, fibroblasts and Schwann cells (Tan *et al*., 2012). The latter study demonstrated by short hairpin RNA knockdown that kindlin‐2 is required for integrin signalling and axonal growth of neurons. Thus, kindlin‐2 is the only isoform endogenously expressed in neurons and plays a role in normal axonal growth. Kindlin‐3 is predominantly expressed by cells of the immune system (Malinin *et al*., 2009; Moser *et al*., 2009; Feigelson *et al*., 2011; Cohen *et al*., 2013; Moretti *et al*., 2013) and recently has been discovered in microglia of the brain (Meller *et al*., 2017).

Kindlin‐1 has been used *in vivo* to promote integrin activation and sensory axonal regeneration in rats. Forced expression of kindlin‐1 (but not the overexpression of the endogenously present kindlin‐2) enhanced the signalling of the integrins that are expressed by DRG neurons. Importantly, kindlin‐1 promoted neurite outgrowth on the axon‐repulsive substrates aggrecan and Nogo‐A (Tan *et al*., 2012). Furthermore, kindlin‐1 counteracted the inhibiting effects of aggrecan on neurite outgrowth of α9 integrin‐transfected DRG neurons *in vitro* (Cheah *et al*., 2016). In accordance with the enhanced outgrowth, the decreased phosphorylation of FAK induced by repulsive substrates was reversed by kindlin‐1 (Tan *et al*., 2012; Cheah *et al*., 2016). Thus, kindlin‐1 overcomes aggrecan‐ and Nogo‐A‐mediated inhibition of integrin signalling and restores DRG neurite outgrowth *in vitro*. Furthermore, after a dorsal root crush injury *in vivo*, forced expression of kindlin‐1 in the DRG enhanced sensory axonal regeneration. In this study, kindlin‐1 treatment using viral vectors resulted in a fairly large number of axons extending towards the spinal cord, while the regenerating axons of the control animals did not pass the axon‐repulsive dorsal root entry‐zone boundary. Consistent with the improved sensory axonal regeneration, kindlin‐1 treatment also improved recovery of thermal sensation after injury (Tan *et al*., 2012). Thus, kindlin‐1 activates integrins that are expressed by DRG neurons and overcomes the inactivation of the axon‐repulsive environment to promote sensory axonal regeneration. In other words, kindlin‐1 overexpression renders integrins less vulnerable to integrin inactivation and thereby restriction of axonal regeneration. Kindlins are subject to regulation by other pathways, although at present this is not well understood (reviewed in Rognoni *et al*., 2016).

#### (c) *Kindlin‐1 and α9 integrin overexpression*

Integrin‐mediated regeneration is most successful when the appropriate integrin is both present and activated. Thus, co‐overexpression of kindlin‐1 and α9 integrin forms a strong stimulus for axonal regeneration in tenascin‐C‐rich areas such as the dorsal root entry zone and spinal cord after a dorsal root crush (Cheah *et al*., 2016). Viral vector‐mediated delivery of both molecules to DRGs indeed resulted in a synergistic effect on sensory axonal regeneration. The α9‐ and kindlin‐1‐overexpressing axons that reached the spinal cord regenerated from the cervical dorsal root at levels C8 to C5 all the way up into the medulla (Cheah *et al*., 2016). Mechanical pressure and thermal sensation in the paw as well as limb proprioception improved after injury in animals that had combined α9 and kindlin‐1 overexpression. Furthermore, electrophysiological recordings demonstrated that sensory pathways from the paw to the dorsal horn of the spinal cord had regrown following injury and α9/kindlin‐1 overexpression. Thus, the combination of α9 and kindlin‐1 leads to robust axonal regeneration of at least 25 mm and partial functional recovery after a dorsal root crush. Furthermore, these results demonstrate that there is a synergistic effect exceeding that of overexpression of α9 (Andrews *et al*., 2009) or kindlin‐1 (Tan *et al*., 2012) alone. Surprisingly, no severe degree of axonal misguidance occurred in this study, with regenerating axons being found mainly in the dorsal column and terminations in the dorsal horn being predominantly in the correct laminae. These results suggest that when activated integrins encounter an appropriate ECM environment, the remaining structures in the CNS can exert guidance effects on the α9/kindlin‐1‐overexpressing sensory neurons.

Taken together, there are various approaches to activate integrins, each with a unique mechanism to promote integrin signalling (Fig. 3). We have reviewed the evidence that stimulation of integrin signalling in injured neurons is a powerful strategy to boost sensory axon regeneration following CNS injury because it can overcome the repulsive molecules that prevent axonal regeneration in the injured spinal cord. To date, the synergistic effects of kindlin‐1 and α9 delivery achieved the longest regeneration observed in the dorsal column pathway by modulating integrin signalling *in vivo* (Cheah *et al*., 2016). Identifying the integrin adhesome is an active field of research and novel integrin activators are therefore continuously being discovered, such as reelin (Lin *et al*., 2016), sema7A (Pasterkamp *et al*., 2003), shank (Lilja *et al*., 2017) and vimentin (Kim *et al*., 2016). The identification of new integrin‐activating molecules also offers opportunities for future regeneration research.

## VII. DEVELOPMENTAL CHANGES IN NEURONAL INTEGRIN LOCALISATION

### (1) Exclusion of integrins from the axon of certain adult central nervous system neurons

Integrins are expressed in developing neurons and have essential roles in the formation of a functional nervous system. They are important for migration (Tate *et al*., 2004; Andressen *et al*., 2005; Marchetti *et al*., 2010), proliferation (Blaess *et al*., 2004; Leone *et al*., 2005), adhesion (Tate *et al*., 2004), differentiation (Tate *et al*., 2004; Andressen *et al*., 2005), axon outgrowth (Sakaguchi & Radke, 1996; Harper *et al*., 2010), axon guidance (Huang *et al*., 2006; Myers *et al*., 2011) and lamination (Georges‐Labouesse *et al*., 1998; Marchetti *et al*., 2010) of neuronal precursor cells of the nervous system. However, during maturation of CNS neurons selective transport mechanisms are set up that send some molecules to dendrites and others to axons (reviewed in Lasiecka & Winckler, 2011; Britt *et al*., 2016; Bentley & Banker, 2016). This selective polarised transport is essential for giving axons a set of molecules and properties appropriate for their function. As part of this general acquisition of polarity, integrins become excluded from CNS axons (Bi *et al*., 2001; Franssen *et al*., 2015). The overall result of these polarity changes is that mature neurons are not able to regenerate, probably due to the absence of various receptors including integrins in their axons.

The distribution of integrins in axons during maturation has been intensively studied, since any treatment involving integrin expression aiming at promoting axon regeneration requires the expressed integrins to reach the axonal compartment and growth cone. By examining localisation of tagged integrins (α6, α9, and β1) *in vivo* in mature and immature sensory, retinal, cortical and red nucleus neurons, a differential ability for integrins to localise within axons became apparent (Andrews *et al*., 2016). Integrins were transported into the still‐developing early postnatal axons of the corticospinal tract, but the investigated α6, α9 and β1 integrins were excluded from mature corticospinal tract and rubrospinal tract axons. High levels of integrins were found in both branches of adult DRG axons and in some RGC axons (Andrews *et al*., 2016). It is tempting to correlate this transport with the ability of immature and sensory axons to successfully sprout and regrow following damage (Bregman & Bernstein‐Goral, 1991; Bates & Stelzner, 1993). In addition and as reviewed earlier, overexpression of α9 integrin in the DRGs indeed stimulated axonal regeneration (Andrews *et al*., 2009; Cheah *et al*., 2016). Integrin‐driven regeneration in the spinal cord and elsewhere will require an intervention to ensure that the molecules are transported into the axons. However, it is not just integrins that are excluded from axons, but many growth‐related molecules, as described below (Section VIII).

### (2) Developmental changes in the integrin transport machinery

**Figure 4:**
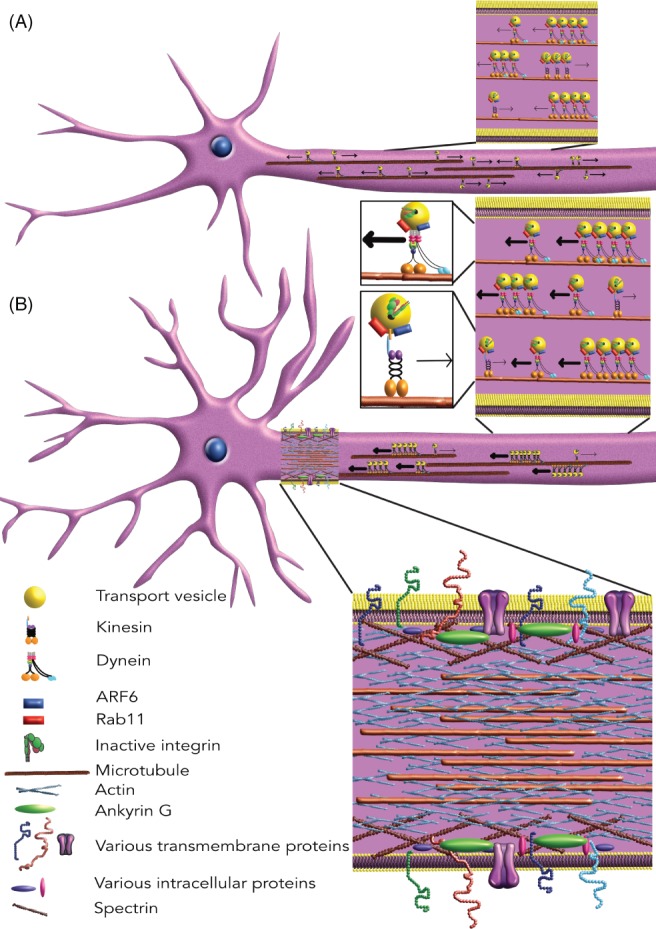
Comparison of immature and mature central nervous system neurons. (A) Immature neurons do not have a fully developed axon initial segment and their axons have been shown to transport integrins both antero‐ and retrograde to an equal extent. Vesicles bound to ADP‐ribosylation factor 6 (ARF6)‐ and Ras‐associated binding (Rab) protein 11 (Rab11)‐GTP are retrograde transported, while vesicles bound to ARF6‐ and Rab11‐GDP move in the anterograde direction. (B) Mature neurons have developed an axon initial segment and are characterised with predominant retrograde axonal transport of integrins.

The exclusion of integrins from the axons of many adult CNS neurons, such as the corticospinal tract, is mediated by the development of selective transport mechanisms that are responsible for neuronal polarity (Fig. 4). Studying integrins provides a good tool to study these mechanisms. Integrin trafficking is well studied in cancer cells, where it was found to be transported in recycling endosomes, which are regulated by small GTPases (Powelka *et al*., 2004). In neurons axonal integrins are mostly transported in Ras‐associated binding (Rab) protein 11 (Rab11)‐ (Caswell *et al*., 2008; Eva *et al*., 2010) and ARF6‐ (Powelka *et al*., 2004; Eva *et al*., 2012) positive recycling endosomes. These GTPases control endosomal targeting and are turned on by GTP exchange factors (GEFs) and turned off by GTP activating proteins (GAPs). Rab11 and ARF6 are responsible for transporting integrins into axons probably as part of a complex with scaffolding molecules, such as the JNK‐interacting protein 3 (JIP3) and JIP4 and kinesin‐ and dynein‐motors (Isabet *et al*., 2009; Suzuki *et al*., 2010; Montagnac *et al*., 2011). In immature neurons there exists anterograde integrin transport, but with maturation there is gradually less anterograde and more retrograde integrin transport in the axon, leading to the exclusion of integrins. In cultured cortical neurons from embryonic day 18 rat pups, expression levels of α5, αV and β1 integrins started to decrease after 7 days in culture and were undetectable in the axon after 14 days (Franssen *et al*., 2015). This exclusion of integrins from the axon coincides with the formation of the axon initial segment (AIS) (Song *et al*., 2009), which plays a part in the exclusion of integrins since disruption of the AIS increased the amount of integrin within mature axons (Franssen *et al*., 2015). The AIS exhibits a dense network of proteins including actin, which can restrict access of molecules to axons by acting as a size filter or by supporting retrograde myosin‐driven transport (Song *et al*., 2009; Lewis *et al*., 2009; Arnold, 2009). There is also a role for actin and modifications of the microtubule cytoskeleton in regulating integrin transport (Franssen *et al*., 2015). However, the main mechanism for exclusion is the gradual change of the transport direction during maturation and the establishment of the AIS. The direction of transport is defined by the activation state of ARF6. ARF6 can be inactivated by its GAP ArfGAP with coiled‐coil, ankyrin repeat and PH domains 1 (ACAP1) and in its inactive state favours anterograde transport (Jackson *et al*., 2000; Dai *et al*., 2004). In turn, active ARF6 favours retrograde transport (Eva *et al*., 2012). Activators of ARF6 are GEFs; two known ARF6 GEFs are cytohesin 2 (ARNO) and plekstrin homology and SEC7 domain‐containing protein (EFA6) (Sakagami *et al*., 2006). Importantly, it has been found that during cortical neuronal maturation ARNO and EFA6 are strongly up‐regulated (Sakagami *et al*., 2006; Franssen *et al*., 2015) and EFA6 localises to the AIS (Eva *et al*., 2017). Both, ARNO and EFA6 are important for the exclusion of integrins from axons (Franssen *et al*., 2015; Eva *et al*., 2017). Interestingly, it has also been found that Rab11 was largely excluded from mature axons, being present at low levels in axons compared to dendrites in primary cortical neurons grown in culture for more than 14 days (Franssen *et al*., 2015; Koseki *et al*., 2017). Overexpression of Rab11 in these neurons permitted integrin transport into the axon and promoted regeneration after laser‐induced axotomy *in vitro* (Koseki *et al*., 2017).

In summary, a developmental switch in the transport of essential growth molecules, such as integrins, results in the exclusion of these molecules from mature CNS axons, likely rendering them unable to regenerate after injury. Interfering with this developmental switch will result in the presence of integrins and other excluded molecules in the axon (Franssen *et al*., 2015; Eva *et al*., 2017; Koseki *et al*., 2017). Based on our observations *in vitro*, we further hypothesise that interfering with this developmental transport switch will lead to increased regeneration after injury.

## VIII. THE LOCALISATION OF OTHER REGENERATION‐ASSOCIATED RECEPTORS

Cell surface receptors are promising targets to promote axonal regeneration (reviewed in Cheah & Andrews, 2016). Rab11‐positive recycling endosomes contain integrins, but also regulate the transport of other regeneration‐associated receptors including tropomyosin receptor kinase receptors (Trks) (Ascaño *et al*., 2009; Lazo *et al*., 2013) and insulin‐like growth factor receptors (IGFRs) (Romanelli *et al*., 2007). The observation that Rab11 vesicles are excluded from the axon *in vitro* (Franssen *et al*., 2015; Koseki *et al*., 2017) is consistent with an *in vivo* study that observed a somatodendritic distribution of Rab11 in the forebrain, cerebellum, thalamus and brainstem (Sheehan *et al*., 1996). Here, we discuss that TrkB and insulin‐like growth factor 1 receptor (IGF‐1R) are also excluded from axons of the adult corticospinal tract and the implications of this for regeneration.

### (1) Tropomyosin receptor kinase B

TrkB is a cell‐surface receptor that can boost the regenerative response of injured neurons. It binds several neurotrophic factors including brain‐derived neurotrophic factor (BDNF), neurotrophin‐3, and neurotrophin‐4. These neurotrophic factors promote neuronal survival and axonal growth and are involved in synaptic plasticity (reviewed in Minichiello, 2009; Park & Poo, 2013; Harrington & Ginty, 2013). Due to the important role of these factors, it may not be surprising that there is a widespread distribution of TrkB in the adult brain (Yan *et al*., 1997). Interestingly, adult corticospinal neurons express TrkB in their cell bodies and dendrites, but not in the axon (Yan *et al*., 1997; Lu, Blesch & Tuszynski, 2001). Furthermore, TrkB and its other family members, TrkA and TrkC, are not up‐regulated after spinal cord contusion (Liebl *et al*., 2001). Consistent with the absence of TrkB in the corticospinal tract, BDNF‐secreting cell grafts in a spinal cord lesion site did not promote axonal regeneration of this motor pathway (Lu *et al*., 2001). Viral vector‐mediated overexpression of TrkB has been shown to result in receptor trafficking into the axon at the level of the subcortical white matter but not further down into the spinal cord (Hollis *et al*., 2009 *b*). These neurons were able to regenerate into BDNF‐secreting cell grafts that were placed into subcortical lesions (Hollis *et al*., 2009 *b*). However, as elaborated above for integrin receptors, additional interventions would be required to enhance the transport of TrkB into the corticospinal tract to promote substantial regeneration after spinal cord injury. In addition, it had been shown in hippocampal slice cultures that the activation state of TrkB correlates with axonal sprouting (Aungst, England & Thompson, 2013). The activation state of Trk receptors may therefore influence the regeneration response as well. Taken together, the absence of TrkB in corticospinal tract axons likely contributes to the restricted axonal regeneration and responsiveness to BDNF treatments after spinal cord injury.

### (2) Insulin‐like growth factor receptor

IGFR is the transmembrane receptor for insulin‐like growth factors (IGFs) and has been shown to promote neuronal survival and outgrowth (reviewed in Sullivan, Kim & Feldman, 2008). Its mechanism of axonal transport is unknown. IGF‐1R has been shown to be essential for the formation of the axon in adult RGCs *in vitro* (Dupraz *et al*., 2013)*,* highlighting its crucial role in promotion of axonal growth. IGF‐1R and insulin receptors were also found to be localised in adult DRGs after injury (Craner *et al*., 2002; Xu *et al*., 2004), with their presence likely correlating with the pro‐regenerative response of these sensory neurons. IGFs play an important role during the development of the corticospinal tract (Arlotta *et al*., 2005; Ozdinler & Macklis, 2006), but IGFRs become excluded from axons during maturation of this motor pathway. More specifically, IGF‐IR is exclusively localised in the somatodendritic compartment of the neurons in layer V motor cortex (Hollis *et al*., 2009 *a*). Consistent with the absence of the receptor in the axonal compartment, corticospinal tract axons were not able to regenerate through IGF‐secreting cell grafts that were transplanted into the lesion after a spinal cord injury *in vivo* (Hollis *et al*., 2009 *a*). Interestingly, the latter study showed that the ceruleospinal and raphespinal axons did regenerate into these grafts. We therefore hypothesise that these two descending motor pathways retained IGFRs in their axonal compartments, but the authors did not examine the receptor expression in these neurons. A recent study took an alternative approach and delivered IGF‐1 together with osteopontin into the sensorimotor cortex in order to promote corticospinal tract regeneration. Viral vector‐mediated delivery of both ligands in the cortex, but not IGF‐1 or osteopontin alone, promoted axonal regeneration approximately 1 mm beyond the lesion site after spinal cord hemisection (Liu *et al*., 2017). The treatment showed the strongest effect on compensatory sprouting from the uninjured side of the spinal cord (Liu *et al*., 2017). The axonal regeneration and sprouting contributed to the improved hindlimb function of the animals after spinal cord injury. The mechanism by which IGF‐1 and osteopontin in the cortex mediate their growth‐promoting effect is unclear. It is possible that both ligands activate their receptors in the somatodendritic domain of cortical neurons, which in turn, activate the PI3K/mammalian target of rapamycin (mTOR) signal transduction cascade. This is sufficient for robust sprouting in the spinal cord, and short‐range regeneration (Liu *et al*., 2017).

Taken together, like integrins, the deficit of TrkB and IGF‐IR in the axons within the corticospinal tract may limit its regeneration. Further investigation is required to determine whether the exclusion of these receptors in corticospinal tract axons also depends on the presence of the axon initial segment as a barrier and whether the same transport vesicles are involved for their transport as for integrins.

## IX. PERSPECTIVES

Integrins are important mediators of axonal regeneration in the injured nervous system. Integrins stimulate axonal regeneration when they are activated and localised at the growth cone to interact with the ECM. In order to use receptors as potential therapeutic targets to promote axonal regeneration, the mechanisms of axonal transport and trafficking need to be better understood. The successful use of activated integrins to promote regeneration of sensory axons leading to recovery of mechano‐ and temperature sensations *in vivo* (Cheah *et al*., 2016) indicates that the overall strategy can be successful. Regeneration of the corticospinal pathway is a key event that is necessary to restore motor control after spinal cord injury. If in addition to integrin activation, the integrin trafficking barrier in descending corticospinal motor neurons could be overcome, then motor recovery could be a surmountable obstacle. Strategies to initiate trafficking to the axonal compartment of the corticospinal tract could therefore be based on: (*i*) overcoming the transport block of the axon initial segment and; (*ii*) stimulation of anterograde transport by modulation of transport vesicles; or (*iii*) adding axonal localisation signals to growth promoting receptors to enter the axon.

## X. CONCLUSIONS

(1) Integrins are localised at the growth cone of immature and regenerating neurons and connect the extracellular and intracellular compartments of the neuron.

(2) Matching the extracellular matrix environment with the appropriate integrins promotes limited axonal regeneration of mature neurons.

(3) Presence of integrins in the axon correlates with the regenerative capacity of neuronal pathways.

(4) Integrins participate in spontaneous axonal regeneration after peripheral nerve injuries.

(5) Axon‐repulsive molecules at the lesion site of spinal cord injuries inactive integrins and thereby inhibit axonal regeneration in the central nervous system.

(6) Stimulation of integrin signalling can overcome the repulsive molecules at the site of injury and promote limited sensory axon regeneration in the central nervous system.

(7) Integrins become excluded from the axon during maturation of most central nervous system neurons and this correlates with the loss of the regeneration ability of mature neurons.

(8) Pioneering work targeting integrins to the axons of mature neurons to promote regeneration serves as a model for other regeneration‐associated receptors that are excluded, such as TrkB and IGF‐1R.

## XI. ACKNOWLEDGEMENTS

This review was funded by a Nathalie Rose Barr award (NRB110) from the International Spinal Research Trust, a grant from the Medical Research Council (G1000864), an ERA‐NET NEURON grant AxonRepair (013‐16‐002) and support from the Hersenstichting Nederland, NWO, and the Laboratory for Regeneration of Sensorimotor Systems at the Netherlands Institute for Neuroscience.
